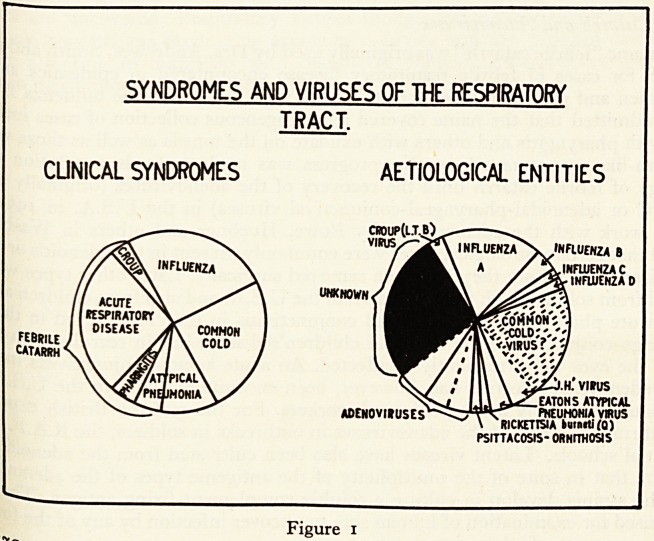# Diseases in Search of Viruses

**Published:** 1958-04

**Authors:** C. H. Stuart-Harris

**Affiliations:** Professor of Medicine, University of Sheffield


### DISEASES IN SEARCH OF VIRUSES

BY

C. H. STUART-HARRIS, M.D., F.R.C.P.

*Professor of Medicine, University of Sheffield**

Everyone agrees that tremendous changes have occurred in the past few years in the field of the infectious diseases. Formidable weapons have been forged both for the treatment and the prevention of bacterial infections. As a result, diphtheria has become almost extinct and the enteric diseases are much reduced, patients with streptococcal disease and with pneumonia less often require hospital treatment, and the burden of acute illness in childhood has considerably lightened. Virus infections have not, however, altered in the same way or to the same extent. Some, such as poliomyelitis, have become a threat to both children and adults. Previously undescribed conditions such as myalgic encephalomyelitis or Royal Free disease, aseptic meningitis with an exanthem and little known syndromes such as herpangina, pharyngo-conjunctival fever and Bornholm disease have achieved a position of interest and even of importance as causes of morbidity. Moreover, the development of new techniques for the cultivation of viruses has led to the realization of a method of control of certain diseases and to a new look in regard to diagnosis. These changes have affected the work of both clinicians and microbiologists, and yet there has also arisen a considerable divergence of outlook between these two groups of medical men. One could almost say that they live in two different worlds.

The virologist, being equipped with new techniques for the laboratory study of viruses, has been more and more excited over the possibility of fundamental discovery concerning the nature of viruses, of their mode of multiplication and of the possibility of restraining their multiplication by chemical substances. Whenever he has occupied his time with human specimens he has found less difficulty in recovering viruses than in classifying the agents thus recovered and in relating their presence in specimens to the particular phase of illness or health existing in those from whom they were derived. There has therefore arisen the phrase of "Viruses in search of disease" which has been applied chiefly to the poliomyelitis-like viruses, to the Coxsackie viruses and to the adenoviruses. One gets the impression that virologists are at times unmoved by the clinical implications of their discoveries and that therefore they are sometimes tempted to urge the use of prophylactic weapons against viruses before it has even become apparent that they are a significant cause of human distress!

The clinician's world is of course familiar to most of you. He moves in an atmosphere of individual illnesses or of family outbreaks where diagnosis and treatment are the paramount needs. He looks on viruses as mysterious microbes very similar to bacteria except in size and blames all diseases of unknown aetiology upon their activity. He is impatient to solve clinical problems in the way that so many problems were solved in the past, namely by first delineating a syndrome sufficiently clearly for it to be called a clinical entity and then by seeking investigation from all possible angles in order to uncover the virus lurking in the patients' interior. Unfortunately clinicians do not always understand the importance of epidemiological studies, the meaning of latent or subclinical forms of virus infection and the significance of virus infections in relation to human ecology (in spite of the prevailing influence of rabbit myxomatosis!) Naturally as a clinician I am sympathetic to the clinician's difficulties, and I am equally conscious of the dangerous path which virology is tempted to pursue. I therefore feel it my duty to try to bring together these two contrasting points of view whenever I

* From an address to the Bristol Medico Chirurgical Society, 8th January, 1958.

can. To do this I must attempt to make the virologist's world a little more easy for clinicians to understand.

*Virus Growth in Tissue Cultures*

Research on animal viruses has characteristically depended upon the introduction of methods of cultivation, each one of which has yielded a certain harvest of knowledge but which has eventually failed to throw fresh light on existing problems. The new look in virus research at the present time stems from the introduction of tissue culture methods by the Boston group of workers centring around Enders at the Boston children's hospital. Helped by the use of antibiotics to control contamination, Enders transformed tissue cultures from their previous position of technical complexity to a method capable of exploration by any laboratory. He was also instrumental in placing the tissue cultures within reach of the low power of a microscope so that simple histological observation of the changes wrought by virus multiplication could be effected.

The two chief ways of preparing tissue cultures for virus cultivation are the explant method where morsels of tissue are embedded in a plasma clot and allowed to develop a halo of growing cells and the roller-tube technique of cells dispersed by trypsinization where the wall of the test-tube becomes covered with a sheet of cells. Virus growth in cells may produce disintegration and cell-death as with poliovirus or else the cells may alter morphologically but remain alive as with the adenoviruses. In both cases the changes observed microscopically are termed cytopathogenic alterations but it is occasionally true that viruses may grow in cells without producing any visible alterations. Cytopathogenic changes form the basis of methods for the titration of virus and of neutralization tests for measuring specific antibodies against the viruses in human or animal sera.

Morphological studies of viruses show a great variation but animal and human viruses are usually composed of small rounded particles as viewed by the electron-microscope. Internal complexity of organization of these elementary bodies is sometimes revealed by methods such as exposure of the virus to enzyme action. Similar internal complexity is revealed by the thin tissue-slice method of electro-micrography perfected by Councilman Morgan and his colleagues in New York. The virus bodies of vaccinia and herpes are readily seen by this method developing in cytoplasmic granular areas within complete or incomplete shells of a surrounding membrane; but in the case of influenza no organized structure is found except at the cell surface. Here the rounded bodies and filamentous forms can be seen escaping from the cytoplasm in great numbers. The development of the virus from the moment of entry into the cell until its release is hidden from view and cannot for some hours be followed even by chemical or serological methods. It seems that the virus particle merges its identity with the cell nucleus or cytoplasm and diverts metabolic activity towards the replication of its own genetic material which resides in its nucleic acid content. Thus the multiplication of viruses is so different from that of bacteria that we are justified in regarding them as a distinct order of organized life, if indeed one can regard them as possessing the essential attributes of living matter.

### SOME COMMON INFECTIONS AND THEIR VIRUSES

Now let us turn to the diseases which either have been in search of viruses until recently or which still are in search of their causative organisms. First let us consider the exanthemata—measles, rubella, chickenpox and herpes zoster:

*The Exanthemata*

Measles virus was transmitted to monkeys some years ago but no one was really convinced that a successful method of laboratory culture had been found until the Boston workers published their study on measles virus in tissue culture. The virus produces an unusual cytological change—the various cells of a human or monkey cell culture merge their identity into a syncytium so that large giant cells are produced. One might in passing mention that giant cells can readily be demonstrated by swabbing the nares or nasopharynx in children with measles and smearing the cellular exudate on slides: this is a useful diagnostic method for measles. But the tissue culture giant cells have permitted the development of work on antibodies during convalescence from infection and the titration of gamma globulin against measles virus. Moreover, Enders has carried the virus in cultures of human amnion, transferred it to chick embryo cultures and is approaching the development of a vaccine against the disease. A more doubtful line of work is the present suggestion that measles is related to dog distemper virus. This arose from the demonstration that most children develop antibodies to distemper virus as they grow up. But the distemper virus produces such distinctive changes in the ferret that it seems to be unlikely that this virus is related to measles and more work on this theme is required. Rubella has so far defied attempts to define its causation. Chickenpox and herpes zoster virus have been grown in tissue cultures but transfer from one culture to the next can only be made by using intact cells, i.e. by grafting. Grinding up the cells prevents the virus from re-entry into new cells so that the relation between virus and host cell must be extremely intimate. Glandular fever has proved much more baffling. No success has been achieved in growing the agent associated with mononucleosis accompanied by a positive Paul Bunnell reaction. Yet a few cases of apparent glandular fever with a negative Paul Bunnell test have yielded Toxoplasma on cultivation. This organism is not of course a virus but the proof that it can cause mononucleosis shows that glandular fever is a syndrome rather than a disease.

### *Alimentary Diseases*

Next the alimentary viruses require consideration. The innocent term "D. and V." obviously includes many unrelated conditions. I personally agree with the suggestion that various coliform bacteria are important causes of infantile gastro-enteritis perhaps especially the form occurring as outbreaks in maternity homes and hospitals. Attempts to demonstrate viruses which cause gastro-enteritis have not been successful, though Gordon in the U.S.A. has transmitted diarrhoea with cell-free filtrates of stools to human volunteers. But none of the welter of viruses found in the stools of healthy and sick children has yet been shown clearly to bear an aetiological relation to gastro-enteritis. Nor has the virus of hepatitis or of homologous serum jaundice been cultivated successfully though again the human volunteer has conclusively proved that both viruses exist in human specimens or excreta. Here then is a big group of alimentary diseases in search of viruses.

### THE ENTERO-VIRUSES (*Orphan and Coxsackie viruses*)

If we pass from consideration of the alimentary diseases to the viruses which inhabit the alimentary tract, the present situation concerning the poliomyelitis-like viruses and the Coxsackie group of viruses forms another problem, this time of viruses in search of disease.

A large family of viruses is now known which consists of many different antigenic species and which have hitherto been grouped either as "orphan" viruses or as belonging to the Coxsackie viruses.

### *Coxsackie Viruses*

The Coxsackie viruses were recovered first of all and were found by Dalldorf by using a method of inoculation of human specimens into suckling mice. The first strains were recovered from the stools of children ill during an outbreak of poliomyelitis in the little town of Coxsackie in the New York State. The viruses would not grow in adult mice or infect monkeys and it was hard to associate them with specific clinical illnesses. Since then a large number of similar viruses with the same inability to attack adult mice yet with pathogenic properties in baby mice has been recovered. Some, now known as Coxsackie A viruses, cause necrosis of striated muscle with resulting flaccid paralysis of the mice, and others (Coxsackie B) cause encephalitis in baby mice but no muscle lesions. Twenty or more serologically different types of Coxsackie A viruses and six variant B viruses have been delineated.

The clinical disease now accepted as being due to Coxsackie A viruses is a trivial fever of infants and children associated with herpes-like vesicles on the palate. This is herpangina as described by Zahorsky in 1920 and it has been found as family outbreaks or larger epidemics in the summer season. Yet a majority of children from whom the Coxsackie A viruses have been recovered have remained clinically well so that infection is inapparent or subclinical. Dalldorf felt that poliovirus infection might in some way be potentiated by Coxsackie virus but could not prove this except that he found both viruses to be present in some faeces. The Coxsackie B viruses are biologically quite distinct. They are accepted as a cause of Bornholm disease and also as a cause of infantile myocarditis. In addition they have been found in faeces or the C.S.F. of some cases of lymphocytic meningitis.

*Echo Viruses*

It is usually difficult to cultivate the Coxsackie viruses in tissue cultures though this has been done with some strains. So when viruses were recovered from tissue cultures inoculated with stools and which would neither infect baby mice nor monkeys, it was realized that yet another group of enteric viruses existed which belonged neither to the polio nor to the Coxsackie group. As no one could associate these viruses with any clinical syndrome they were termed "orphans" or to give them their full title—the ECHO (enteric, cytopathogenic, human orphan), viruses. If one were to call the Coxsackie viruses the first cousins of polio, one ought to call the ECHO strains poliovirus second cousins. Already sixteen serologically different types are known.

*The Lymphocytic Meningitis Syndrome*

Wallgren in 1924 drew attention to lymphocytic meningitis as an important syndrome of multiple aetiology and called the non-bacterial forms "aseptic meningitis". With increased study of outbreaks of poliomyelitis many cases of non-paralytic disease have been recognized whose clinical picture conforms to Wallgren's description. However, when an aetiological study of lymphocytic meningitis is attempted, it has been the experience of many workers that the polioviruses will only account for a third or less of all the cases. Some few are due to mumps (with or without parotitis), and fewer still to lymphocytic choriomeningitis virus derived probably from house-mice. In Sheffield Drs. Tyrrell, Clarke and Heath have studied an outbreak of lymphocytic meningitis which appears to have been due to a single species of enterovirus. The epidemic was part of a general European conflagration of which the first signs appeared in 1955 in the Marche province of Italy.

From the throat and also from the stools of the patients an Italian virologist called Archetti readily recovered strains of a virus which he regarded as a Coxsackie A virus. The epidemic was not reported at the time. In 1956, first in East Anglia, then in Belgium, the Netherlands and many parts of Germany and then in the Midlands of England, aseptic meningitis occurred as an epidemic, chiefly affecting children but now accompanied by a definite rash in about one fifth of the cases. The rash is a blotchy or morbilliform eruption resembling roseola infantum. In Sheffield, Dr. Heath had been working with a modified strain of poliovirus type 2 in tissue culture and had learnt the appearances of a feebly virulent strain in monkey kidney cells. When throat swabs and stools from this "rash disease" were inoculated into kidney cell cultures, somewhat similar appearances were seen, suggesting feeble virus activity. Many strains of a virus were readily cultivated but for some time these could not be identified. Then one day some baby mice were inoculated with the tissue cultures and much to our surprise the typical appearances of a Coxsackie A virus were obtained. Yet direct inoculation of stools or throat swabs into mice failed to produce any illness. The viruses were next compared with others received from the East Anglian and other European outbreaks and found to be serologically identical. Ultimately they were proved to be examples of a virus hitherto called an ECHO type 9, of which the prototype strain was recovered from the stool of a healthy child in Cincinatti. It thus seems that the ECHO 9 is really a Coxsackie A virus and is one cause of lymphocytic meningitis.

Meanwhile the story of aseptic meningitis has not ended with this ECHO 9 experience. ECHO viruses types 4 and 6 have also caused outbreaks of aseptic meningitis. Yet we still encounter cases from whom no viruses can be recovered either from stools or C.S.F. The problem of lymphocytic meningitis is thus still a task for the future which is made worse by the multiplicity of the virus species concerned in its aetiology. The enteroviruses like the Salmonellae seem perfectly adapted as human parasites and they furnish a vast and perhaps a growing challenge. For just as poliomyelitis has become an epidemic disease largely because of the postponement of the initial infection with poliovirus from infancy to a later age, so the enteroviruses may cause clinical disease if they first encounter the child after the period of infancy has passed.

### RESPIRATORY DISEASES IN SEARCH OF VIRUSES

The number of known viruses which can cause respiratory disease is growing and yet a large proportion of the total of acute respiratory disease remains of undetermined aetiology. The four basic syndromes of influenza, the common cold, atypical pneumonia and acute undifferentiated respiratory disease still furnish a reasonably complete description of the clinical counterparts of the various non-bacterial respiratory diseases. These clinical syndromes are depicted on the left in the diagram and the known aetiological entities on the right (*Figure* 1).

Figure 1

### *Influenza*

Influenza is largely accounted for by infection with the two groups of influenza viruses A and B. Influenza C has not so far occurred in epidemic fashion but appears to be a cause of sporadic febrile respiratory illness. Influenza D is the name given to a virus recovered originally in Japan from cases of pneumonitis in the neonatal period. Until last year it had only been found to be associated with sporadic illness but an epidemic occurred in Vladivostok in 1956 and this was apparently regarded as clinical influenza. The D virus differs a good deal from the other influenza viruses because of its destructive effect on cells in tissue culture and by reason of its recovery from wild mice. Nevertheless, it produces agglutination of red cells and has other qualities justifying inclusion in the group of viruses known as "myxovirus" which includes influenza, mumps and Newcastle disease virus of fowls.

*Croup*

A newly recovered haemagglutinating virus also with antigenic relationship to mumps virus is that recovered from cases of acute obstructive laryngo-tracheo-bronchitis (Croup) in 1956 simultaneously by Chanock in the U.S.A. and Beale in Toronto. Dr. Clarke is working with the L.T.B. virus in Sheffield and is endeavouring to find out whether cases of acute respiratory disease in adults are due to this virus or whether it is only a cause of illness in babies.

*Atypical Pneumonia*

Numerous viruses are associated with the atypical pneumonia syndrome, but that form of the disease in which serum agglutinins develop that are active against red cells in the cold (cold agglutinin antibody) has until recently been regarded as of unknown origin. Now, however, fresh evidence has been obtained by Boston workers, associating the disease with the virus recovered by Eaton during the war. One still feels that atypical pneumonia is not a numerically common form of acute disease of the respiratory tract.

*Febrile Catarrh and Adenoviruses*

The name "febrile catarrh" was originally used by Drs. Andrewes, Smith and myself in 1938 for cases of febrile respiratory disease encountered in epidemics amongst servicemen and particularly in recruits, which were not due to the influenza A virus. It was admitted that the name covered a heterogeneous collection of cases including some with pharyngitis and others with exudate on the tonsils as well as those with an influenza-like symptomatology. No progress was made towards a solution of the aetiology of febrile catarrh until the recovery of the adenoviruses (originally termed the APC or adenoidal-pharyngeal-conjunctival viruses) in the U.S.A. in 1953. The earliest work with the adenoviruses by Rowe, Huebner and others in Washington showed that certain serological types were commonly present in the adenoids or tonsils of children from whom they had been removed surgically. Later other types were recovered from soldiers with febrile catarrh in the U.S.A. and also from children affected by an acute pharyngitis. An associated conjunctivitis in the children led to the term "pharyngo-conjunctival fever" for the children's disease but in recruits with febrile catarrh the eyes were frequently unaffected. An acute kerato-conjunctivitis in adults due to adenovirus infection has, however, been encountered both in the U.S.A. and in Britain particularly among shipyard workers. For the rest the British experience has confirmed the role of the adenoviruses in outbreaks in soldiers, the R.A.F. and in residential schools. Latent viruses have also been cultivated from the adenoids. It is fortunate that in spite of the multiplicity of the antigenic types of the adenoviruses, all of the strains develop in culture a soluble complement-fixing antigen. The latter can be used for examination of human sera to uncover infection by any of the fourteen or more serotypes of adenoviruses now known.

In the experience at Sheffield even after exclusion of the cases of influenza or of adenovirus infection, a substantial number of cases of acute respiratory disease remain of undetermined aetiology. Much the same can be said of endemic acute respiratory disease in the civilian population and it is now agreed by most workers that the adenoviruses do not appear to account for a large proportion of the burden of sickness due to respiratory tract infections.

### *The Common Cold*

When we turn to the common cold, the aetiological situation has taken a new look since the description of the virus of Mogabgab. This was first recovered by a group of U.S. Naval Medical Officers who tested throat washings from patients with various acute febrile respiratory disorders in tissue culture. Another strain of this virus was recovered in 1956 by Price from nurses of the Johns Hopkins Hospital suffering from common colds. Price has recently claimed that this virus, which he calls J-H virus, was a cause of 30 per cent. of the colds at a home for babies and infants, and he has also prepared a vaccine from the virus which has some protective effect. The M.R.C. Common Cold Research Unit is testing the J-H virus in human volunteers at Salisbury. I cannot tell you more of this work except to say that the J-H virus will grow in tissue cultures in Britain but that it is a difficult agent to study. Even if it does account for one-third of all the colds from which we suffer, we are still without any clue to the cause of the remainder. Perhaps the common cold is a syndrome due to several distinct agents as is the case with the other forms of acute respiratory disease.

### *The Future*

I will close this lecture by asking for patience to be exerted by those who seek to know more about the many acute diseases of the respiratory tract which are still in search of their causative viruses. Progress though slow is nevertheless steady and so long as laboratory workers are not hampered by insistent demands upon their time for diagnostic work, they will certainly succeed in a further clarification of the present obscure position. The almost bewildering complexity of the aetiological situation is at times discouraging particularly when virus species existing in many different antigenic varieties are uncovered. Respiratory viruses though less diverse than the Coxsackie agents are nevertheless fascinating agents to cultivate and the clinician can help by drawing attention to outbreaks with distinctive clinical characteristics and which appear to merit intensive laboratory study.

## Figures and Tables

**Figure 1 f1:**